# Creatine-induced activation of antioxidative defence in myotube cultures revealed by explorative NMR-based metabonomics and proteomics

**DOI:** 10.1186/1550-2783-7-9

**Published:** 2010-02-04

**Authors:** Jette F Young, Lotte B Larsen, Anders Malmendal, Niels Chr Nielsen, Ida K Straadt, Niels Oksbjerg, Hanne C Bertram

**Affiliations:** 1 Department of Food Science, Faculty of Agricultural Sciences, University of Aarhus, Denmark; 2 Center for Insoluble Protein Structures (inSPIN), Interdisciplinary Nanoscience Center (iNANO), Aarhus, Denmark; 3 Department of Chemistry, Aarhus University, Langelandsgade 140, DK-8000 Aarhus C, Denmark

## Abstract

**Background:**

Creatine is a key intermediate in energy metabolism and supplementation of creatine has been used for increasing muscle mass, strength and endurance. Creatine supplementation has also been reported to trigger the skeletal muscle expression of insulin like growth factor I, to increase the fat-free mass and improve cognition in elderly, and more explorative approaches like transcriptomics has revealed additional information. The aim of the present study was to reveal additional insight into the biochemical effects of creatine supplementation at the protein and metabolite level by integrating the explorative techniques, proteomics and NMR metabonomics, in a systems biology approach.

**Methods:**

Differentiated mouse myotube cultures (C2C12) were exposed to 5 mM creatine monohydrate (CMH) for 24 hours. For proteomics studies, lysed myotubes were analyzed in single 2-DGE gels where the first dimension of protein separation was pI 5-8 and second dimension was a 12.5% Criterion gel. Differentially expressed protein spots of significance were excised from the gel, desalted and identified by peptide mass fingerprinting using MALDI-TOF MS. For NMR metabonomic studies, chloroform/methanol extractions of the myotubes were subjected to one-dimensional ^1^H NMR spectroscopy and the intracellular oxidative status of myotubes was assessed by intracellular DCFH_2 _oxidation after 24 h pre-incubation with CMH.

**Results:**

The identified differentially expressed proteins included vimentin, malate dehydrogenase, peroxiredoxin, thioredoxin dependent peroxide reductase, and 75 kDa and 78 kDa glucose regulated protein precursors. After CMH exposure, up-regulated proteomic spots correlated positively with the NMR signals from creatine, while down-regulated proteomic spots were negatively correlated with these NMR signals. The identified differentially regulated proteins were related to energy metabolism, glucose regulated stress, cellular structure and the antioxidative defence system. The suggested improvement of the antioxidative defence was confirmed by a reduced intracellular DCFH_2 _oxidation with increasing concentrations of CMH in the 24 h pre-incubation medium.

**Conclusions:**

The explorative approach of this study combined with the determination of a decreased intracellular DCFH_2 _oxidation revealed an additional stimulation of cellular antioxidative mechanisms when myotubes were exposed to CMH. This may contribute to an increased exercise performance mediated by increased ability to cope with training-induced increases in oxidative stress.

## Background

Creatine is predominantly situated in skeletal muscle, and originates from both endogenous *de novo *synthesis and exogenous sources, which are mainly animal products [1]. Creatine and its phosphorylated form are well recognized as key intermediates in the energy metabolism of muscle fibres. Supplementation of creatine has been widely used among athletes as a means for increasing muscle mass and muscle strength and muscle endurance [2-4], but also for elderly people creatine supplementation, seems to enhance muscle strength [5]. The rationale behind CMH supplementation is to increase the content of creatine phosphate in the muscle, and several studies have also shown that the creatine content of the muscle is increased [6], and the majority of this is as creatine phosphate [1,2]. However, creatine supplementation has also been reported to trigger the expression of insulin like growth factor I (IGF-1) in young adults [7], increase fat-free mass [8] and improve cognition in the elderly [9]. Some of these findings have been supported by mechanistic studies in various muscle cell cultures, where IGF-1 [10], myogenesis [11] and protein synthesis [10,12,13] were increased, and also a more explorative approach using microarrays on muscle biopsies from creatine supplemented individuals revealed cytoskeleton remodelling, protein and glycogen synthesis regulation, as well as cell proliferation and differentiation [8]. Other techniques such as proteomics and metabonomics may reveal additional insight into some of the biochemical effects of creatine supplementation at the protein and metabolite level.

High-resolution ^1^H nuclear magnetic resonance (NMR) spectroscopy is a well-established analytical technique for metabolic fingerprinting of biofluids and various tissues and has also been used for elucidating the metabolic effects of dietary factors in both humans [14-17], animals [18-20], and also in cell cultures [21]. These studies have demonstrated that NMR-based metabonomics is extremely efficient in detecting endogenous and exogeneous metabolic perturbations. However, while being capable of identifying biomarkers and metabolic perturbations, the metabolic network responsible for the perturbations can only be hypothesised. Proteomics displays protein products as a result of gene expression and efficiency of translation, and has been used to separate and identify differentially regulated proteins in response to various treatments of cultured cells [22,23] and muscles [24]. Linking information obtained from metabolic fingerprinting with proteomics would pave the way for obtaining a better understanding of the primary pathways involved in perturbations associated with CMH supplementation. In this study we have for the first time examined and integrated the NMR metabolite profile and the proteomic profile of myotubes in the presence and absence of creatine supplementation in a systems biology approach.

## Methods

### Muscle Cell Culture

Myotube cultures were established from a mouse myoblast line (C2C12) originally derived from a thigh muscle [25] (American Type Culture Collection, Manassas, VA). A clone from this cell line, which effectively fused and formed myotubes, was isolated [26]. The clone was grown in 80 cm^2 ^culture flask in 10 mL of medium consisting of Dulbecco's modified Eagle's medium (DMEM), 10% (vol/vol) fetal calf serum (FCS), and supplemented with 1% antibiotics giving 100 IU/mL penicillin, 100 μg/mL streptomycin sulfate, 3 μg/mL amphotericin B, and 20 μg/mL gentamycin (growth medium). Cells were maintained in an atmosphere of 95% air and 5% CO_2 _at 37°C. Prior to confluence, cells were harvested in 0.25% trypsin and sub-cultured into 80 cm^2 ^culture flasks or 96 well plates. Cells were grown to confluence in growth medium, and left to fuse in medium containing 4% FCS (differentiation medium) without phenol red. Following approximately 6 days, the cultures contained differentiated multinuclear myotubes and were ready for experimental use. Culture medium was changed every other day throughout the culture period.

### Myotube treatment and sampling for proteomics and metabonomics

For 24 hours the fully differentiated myotubes were cultured in the presence or absence of 5 mM creatine monohydrate (CMH) in the differentiation medium. The treatment and controls were performed in triplicate. Cells were washed in PBS and harvested in 10 ml phosphate buffered saline (PBS) by scraping the flask and mixed thoroughly. The protein content of the cell suspensions was analyzed by the bicinchoninic acid assay (BCA) (BioRad). Five aliquots of 200 μL of each of the triplicates were centrifuged at 6.000 × g for 5 min at 4°C. The cell pellet was kept at -80°C for proteome analysis. The remaining approximately 9 mL was centrifuged at 1000 × g for 10 min at 4°C. The pellet was washed in 1 mL D_2_O including 0.9% NaCl, centrifuged at 6.000 × g for 5 min and the pellet was kept at -80°C for metabonome analysis.

### Two-dimensional gel electrophoresis (2-DGE)

The stored cell pellets were thawed, and 100 μL of lysis buffer (6 M urea, 2 M thiourea, 1.5% (w/v) pharmalyte, 0.8% (w/v) 3-[(3-cholamidopropyl) dimethylammonio]-1-propansulfonate (CHAPS), 1% (w/v) dithioerythritol (DTE) in water) was added to triplicate samples. After incubation for 2 h at room temperature, the desired amount of protein from the two aliquots of each sample was combined and further diluted in a rehydration buffer to a final volume of 185 μL. The rehydration buffer consisted of the same substances, in same concentrations as the lysis buffer, but with pharmalyte (5 μL/mL) instead of 1% DTE. For analytical gels subjected to image analysis, a volume of the lysed cell fraction corresponding to 50 μg protein was applied. For preparative gels used for mass spectrometry (MS) analysis a volume corresponding to 125 μg protein was applied. The lysed cells were analyzed in single 2-DGE gel sets consisting of 6 gels representing the three biological replicates of either control cells or CMH treated cells. The first dimension of protein separation was carried out in immobilized 11 cm IPG strips (pH 5-8), whereas 12.5% Criterion gels (BioRad) were used for the second dimension. Running conditions for the 2-DGE gels were essentially as described earlier [27]. Analytical gels were silver stained according to Lametsch and Bendixen [27], whereas preparative gels were stained according to Shevchenko et al.[28].

### In gel digestion, desalting and concentration of protein spots

Protein spots of significance were subjected to in-gel digestion by addition of trypsin essentially as described by Jensen et al. [29]. Custom-made chromatographic columns were used for desalting and concentration of the peptide mixture prior to MS analysis as described by Lametsch et al. [30]. The peptides were eluted in 0.5 μL matrix solution (15-20 g/L of α-cyano-4-hydroxycinnamic acid in 70% acetonitrile) directly onto the MALDI target plate.

### Identification of myotube proteins by MALDI-TOF mass spectrometry

Mass spectra were obtained using a Bruker Ultraflex MALDI-TOF tandem mass spectrometer in reflection mode. A peptide calibration standard (0.2 μl) containing seven standard peptides ranging in molecular mass from 1046.54 to 3147.47 Da was spotted separately onto the MALDI target plate. The ion accelerating voltage was 25 kV with a delay time of 40 ns. The laser frequency was 50 Hz and 200 laser shots were accumulated for each spectrum. Proteins were identified by peptide mass fingerprinting (PMF) by mass searches in the database Swiss Prot (Swiss Institute of Bioinformatics, Genève, Switzerland) using the search program Mascot (Matrix Science, Boston, USA). In this program the experimental mass value, obtained from MS, is compared with calculated peptide masses from a database. A scoring algorithm is used to identify the closest match. Significant protein identifications (protein scores above 60, P < 0.05) were reported, and manually verified.

### Image analysis

The 2-DGE gels were photographed by a Vilber Lourmat digital camera (ImageHouse, Copenhagen, Denmark) equipped with Gel Pro analyzer software. The gel spots were detected and quantified using ImageMaster 2D platimum software (Amersham Pharmacia Biotech, Uppsala, Sweden). After initial analysis using automated spot detection and segmentation, all images were manually checked and the spots were matched by comparing the relative positions of the individual spots on each gel, which reduced the number of spots used in the further analysis. The spots were quantified by adding the pixel intensities within the spot boundary, and the spot volumes were calculated. To overcome gel-to-gel variations in spot intensities due to technical variations related to the staining procedure, the relative spot volumes were calculated for each separate spot on the gels and these values were used in the further data analysis.

### NMR measurements

Cells were extracted prior to NMR measurements using a dual methanol/chloroform extraction. The culture dishes were placed on liquid nitrogen and cells were added 2 mL of cold chloroform/methanol (1:1, vol/vol). The cells were homogenized using an electrical homogenizer, and centrifuged for 20 min at 1300 g at 4°C. After centrifugation the supernatants were collected and the pellets were resuspended with 1 mL of chloroform/methanol, centrifuged, and the supernatants were collected. The supernatant was washed with 1 mL ice-cold water, and the water phase was removed and added to the pellet. Two mL of water was added, the pellet was centrifuged, the supernatant was freeze-dried and subsequently dissolved in 0.6 mL D_2_O containing 0.5 mM sodium trimethylsilyl-[2,2,3,3-^2^H_4_]-1-propionate (TMSP), and analyzed by ^1^H NMR.

The NMR measurements were performed at 300 K on a Bruker Avance 400 NMR Spectrometer (Bruker BioSpin), operating at a ^1^H frequency of 400.13 MHz, and equipped with a standard 5-mm HX inverse probe. One-dimensional ^1^H NMR spectra were obtained using a single 90° pulse experiment, solvent suppression was achieved by irradiating the solvent peak during the relaxation delay of 2 s. A total of 128 transients of 8 K data points spanning a spectral width of 24.03 ppm were collected. An exponential line-broadening function of 1 Hz was applied to the free induction decay (FID) prior to Fourier transform (FT). All spectra were referenced in chemical shift value to the TMSP signal at 0 ppm. The ^1^H NMR spectra in the 10.0-5.0 and 4.5-0.5 ppm regions were subdivided into 0.005 ppm integral regions and integrated, reducing each spectrum into 616 independent variables. The reduced spectra were normalized to total intensity to remove any concentration effects.

### DCFH_2 _oxidation analysis

Differentiated myotubes in 96 well plates were analyzed as described earlier [31]. Briefly, myotubes were pre-incubated with different concentrations of CMH (0.04-10 μM) for 24 h. Myotubes were then washed and loaded with 10 μM 2',7'dichlorodihydroflourescein diacetate (Molecular Probes, Inc. Eugene, OR) (H_2_DCF-DA) for 2 h at 37°C (95% air, 5% CO_2_) washed again, 100 μM H_2_O_2 _was added and intracellular DCFH_2 _oxidation was determined by fluorescence from 2,7-dichloroflourescein (DCF) at excitation and emission wavelengths of 490 and 515 nm, respectively, at 37°C with a microtiter plate reader (Synergy 2, BioTek Instruments Inc., Vermont, USA). Data is presented as average of 12 replicate wells after background correction.

### Data analyses

Multivariate data analysis was performed using the Unscrambler software version 9.2 (Camo, Oslo, Norway). Partial least squares-discriminant analysis (PLS-DA) was performed on the metabonomic and the proteomic data to explore intrinsic biochemical dissimilarities between control cells and CMH treated cells. For the metabonomic data, the NMR signals were used as continuous X-parameters, while the treatment consisted the discriminant regressors (control = 0, treated = 1). For the proteomic data, the relative spot volumes obtained by image analysis of the 2-DGE gels were used as continuous X-parameters. Protein spots contributing least to the PLS-DA models were removed by Jack-knifing [32] through variable selection until an optimal calibrated and validated model was achieved, and based on the remaining spots significant (P < 0.05) regression coefficients were identified using the uncertainty test. For elucidation of correlations between metabonomic and proteomic data, a PLS-2 regression was carried out with NMR variables as X and proteomic spots identified as significant from the D-PLS model as y-variables. A students' t-test was carried out to compare the concentrations of each myotube protein in the triplicate controls and CMH treated C2C12 cells. A two-tailed paired t-test was used with a 0.95% confidence interval.

## Results

**Figure 1:**
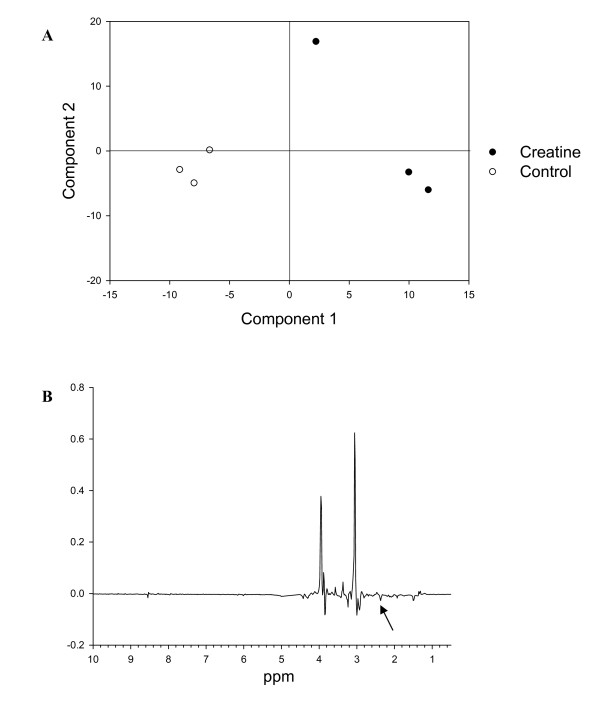
**PLS-DA scores plot of NMR-based metabonomic data**. (A) PLS-DA scores plot from analysis of NMR-based metabonomic data obtained on extracts of control (open circles) and creatine monohydrate (CMH) treated C2C12 muscle cells (closed circles), (B) the X-loadings of the PLS-DA. The dominating signals at 3.04 and 3.94 ppm are ascribed to CH_3 _and CH_2 _in creatine, respectively. The arrow shows a signal at 2.40 ppm, which was also found to be significant in the discrimination of control and CMH-treated cells. The 2.40 ppm signal is tentatively assigned to malate.

**Figure 2:**
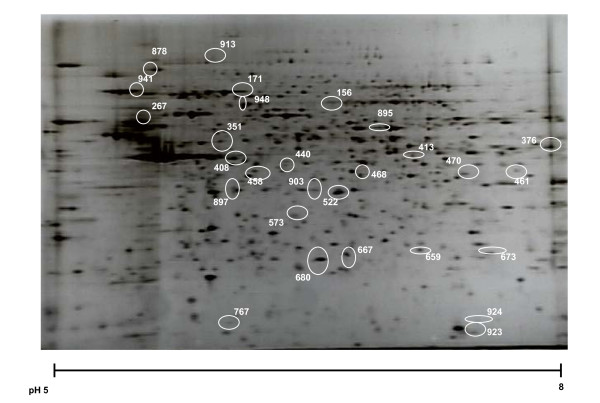
**Proteomic profile of myotubes**. Proteomic profile of myotubes as analyzed by 2-DGE visualized by silver staining. The positions of protein spots identified to be significantly different in controls and in creatine monohydrate-treated myotubes by PLS-DA of 2-DGE proteomics data are indicated.

Myotubes exposed to creatine clearly changed their overall metabolic profile as reflected in the PLS-DA on the NMR-based metabonomic data (Figure 1A), where the discrimination between the control myotubes and CMH treated myotubes was evident. The loading plot (Figure 1B) revealed that signals at 3.04 ppm and 3.94 ppm dominates the discrimination, and this can be ascribed to a higher content of creatine in the treated cells, confirming the expected increased incorporation of creatine into the myotubes. The myotube protein expression in response to creatine was analyzed by proteomics using 2-DGE. An obtained proteomic profile of myotube extracts is shown in Figure 2.

After the manual check of the automatically assigned number of spots, a total of 584 protein spots were annotated by the image analysis and used in the further statistical analysis. By PLS-DA, 28 proteins were found to be differentially expressed when comparing CMH-treated myotubes with the control myotubes (results not shown). The significance of the spots identified by the PLS-DA was further tested by statistical t-test (Table 1). Of the 28 protein spots in the PLS-DA model, 20 of these were found to be either significantly different (P < 0.05) or exhibited tendency to be significantly different (P < 0.1) by the t-test. Accordingly, the t-test confirms that the intensities of the majority of the spots identified by PLS-DA are considerably affected by CMH treatment. Of these, 13 were up-regulated by CMH treatment, while 7 were down-regulated. This shows, as probably expected, that CMH stimulates the expression of more proteins than it down-regulates. The spots which were identified by the t-test to be differentially expressed in the myotubes in response to CMH treatment were cut out from the gels, and subjected to MALDI-TOF MS analysis using peptide mass fingerprinting. Those protein spots which were identified by MS are listed in Table 2. The identified proteins include vimentin, malate dehydrogenase, peroxiredoxin, thioredoxin dependent peroxide reductase, 75 kDa and 78 kDa glucose regulated protein precursors.

**Table 1:** Relative protein spot volumes of spots identified by PLS-DA

Spot ID	Mean C	SD	Mean CMH	SD	n	C→CMH^1^	P-value
156	0.140	0.042	0.271	0.005	3	↑	0.028
171	0.182	0.027	0.138	0.022	3	↓	0.004
267	0.309	0.248	0.811	0.233	3	↑	0.019
376	0.362	0.169	0.109	0.010	3	↓	0.120
408	0.400	0.072	0.380	0.165	3	↓	0.828
413	0.058	0.011	0.0716	0.002	3	↑	0.113
440	0.048	0.004	0.077	0.010	3	↑	0.042
458	0.118	0.003	0.102	0.002	3	↓	0.015
461	0.051	0.008	0.069	0.006	3	↑	0.134
483	0.072	0.005	0.087	0.004	3	↑	0.021
515	0.192	0.027	0.255	0.016	3	↑	0.079
522	0.410	0.008	0.587	0.081	3	↑	0.073
573	0.079	0.008	0.135	0.004	3	↑	0.002
659	0.091	0.005	0.107	0.005	3	↑	0.115
667	0.140	0.005	0.170	0.012	3	↑	0.038
673	0.140	0.027	0.187	0.006	3	↑	0.086
680	0.255	0.009	0.302	0.004	3	↑	0.006
767	0.062	0.005	0.040	0.012	3	↓	0.030
878	0.277	0.086	0.094	0.025	3	↓	0.055
895	0.175	0.011	0.114	0.016	3	↓	0.011
897	0.181	0.049	0.085	0.011	3	↓	0.066
900	0.087	0.008	0.048	0.011	3	↓	0.025
903	0.068	0.020	0.152	0.028	3	↑	0.086
923	0.070	0.018	0.153	0.031	3	↑	0.038
924	0.029	0.006	0.064	0.011	3	↑	0.015
941	0.566	0.184	0.078	0.134	3	↓	0.114
948	0.080	0.020	0.120	0.008	3	↑	0.126
951	0.047	0.021	0.045	0.024	3	↓	0.9

^1^, direction of change of relative spot volume in samples in relation to CHM treatment (C, data from control cells; CMH, data from CMH treated cells).

**Table 2:** Proteins from myotubes identified by MALDI-TOF MS of spots after 2-DGE.

Spot id	Protein	Sequence coverage^a^	Matched peptides^b^	Score^c^	Theo. pI^d^	Theo. Mw^e ^(kDa)	Access key^f^
High in CMH							
267	Vimentin	37	21	189	4.9	54	P20152
522	Malate dehydrogenase - cytoplasmic	21	6	65	6.2	37	Q6PAB3
667	Peroxiredoxin-4	26	6	73	6.8	31	O08807
680	Thioredoxin dependent peroxide reductase	45	9	98	5.9	28	P20108
High in Controls							
171	GRP75, 75 kDa glucose regulated protein precursor	16	10	76	5.8	74	P38674
941	GRP78, 78 kDa glucose regulated protein precursor	24	16	120	4.9	72	P06761

^a^, The minimum coverage of the matched peptides in relation to the full-length sequence.

^b^, The number of matched peptides in the database search.

^c^, Score of the Mascot search.

^d^, Theoretical pI of the full length protein.

^e^, Theoretical molecular mass (Mw) of the full length protein.

^f^, Primary accession key in the SWISS-PROT database.

**Figure 3:**
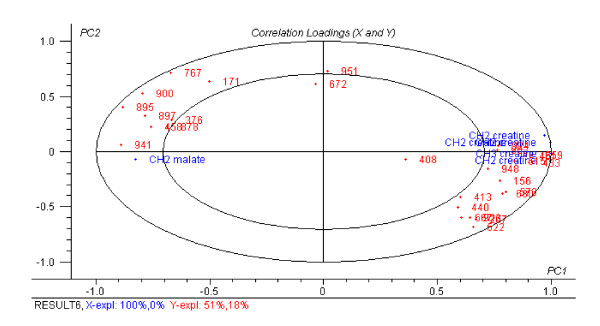
**Correlation loading plot of PLS2 (X: NMR variables, Y: selected proteomic spots)**. Correlation loading plot (1^st ^and 2^nd ^PLS component) of PLS2 using NMR variables as X and selected proteomic spots as Y. Jack knifing has been applied to eliminate insignificant variables. The inner and outer ellipses refer to 50 percent and 100 percent explained variance in X and Y, respectively. The validated explained variances are 100%/0% for X and 51%/18% for Y, the 1^st ^and the 2^nd ^component, respectively.

Moreover, in order to investigate the relationship between the proteomic spots, identified by the PLS-DA model and the metabolite profile of the myotubes, a PLS2 regression was carried out between the NMR metabolite profile and the 28 differentially regulated spots. Figure 3 shows the correlations loadings for variables selected from jack-knifing of this PLS-2 regression. Proteomic spots found to be up-regulated with CMH are clearly positively correlated with the NMR signals from creatine (3.05; 3.95-3.99 ppm), while proteomic spots found to be down-regulated with CMH are negatively correlated with these NMR signals. Noticeably, a NMR signal at 2.39 ppm, which is tentatively assigned to malate, is positively correlated with the proteomic spots down-regulated by CMH.

**Figure 4:**
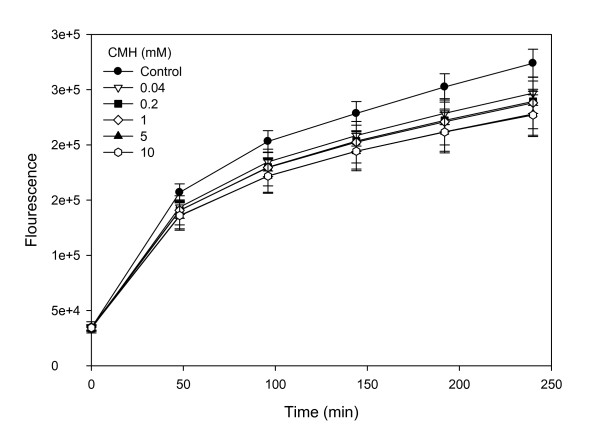
**Intracellular oxidation of 2,7-dichloroflourescein**. Oxidation of intracellular 2,7-dichloroflourescein in myotube cultures exposed to 100 μM H_2_O_2 _after pre-incubation with increasing amounts of creatine monohydrate (CMH) for 24 h.

The results from the proteomic data indicate an antioxidative effect of CMH on the cells as two thioredoxin reductases (peroxiredoxin-4 and thioredoxin dependent peroxide reductase) were up-regulated. On the basis of this, the overall intracellular antioxidative capacity was analyzed in myotubes after pre-incubation with CMH for 24 h. The protective effect of CMH pre-incubation was supported by a reduced intracellular DCFH_2 _oxidation with increasing concentrations of CMH (Figure 4).

## Discussion

The identified differentially regulated proteins (Table 2) are related to different cellular functions. Malate dehydrogenase is central in the energy metabolism, GRP75 and GRP78 are glucose regulated stress proteins, the filament protein vimentin is involved in maintaining cell integrity, and perturbation of the antioxidant defence system is indicated by peroxiredoxin-4 and thioredoxin dependent peroxide reductase.

The reason why malate dehydrogenase is elevated in creatine treated cultures is not known. However, we speculate that increased re-synthesis of glycogen is involved following treatment with CMH. This is based on the following considerations. In muscle creatine phosphate is an available energy source for muscle contraction during anaerobic conditions:

This reaction is under the control of creatine phosphokinase. Addition of CMH increases intra cellular concentrations of creatine (Figure 1) and this in turn will force the equilibrium to the right resulting in a higher level of creatine phosphate and ADP. Reduced ATP and increased ADP will increase the ratio of ADP:ATP which increases the rate of glycogenolysis. Thus, to restore ATP glycogen is degraded causing an elevated intracellular glucose level, which in the present study was indicated by down regulation of the glucose regulated protein precursors GRP75 and GRP78, both of which has been shown to increase with glucose starvation in the cell [33].

Following ATP restoration, glyconeogenesis is stimulated (by ATP). The substrate for the re-synthesis of glycogen is oxaloacetate and in the mitochondria oxaloacetate is converted to malate in order to enable the transport to the cytoplasm. Malate is then oxidised to oxaloacetate in the cytoplasm and this oxidation process is catalyzed by malate dehydrogenase. Oxaloacetate is then available as substrate for glycogen re-synthesis. Increased expression of malate dehydrogenase in CMH supplemented myotubes together with reduced intracellular content of the reaction substrate malate as detected by the NMR signal at 2.39 ppm. (Figure 3) support the assumptions above.

Thus, the data related to cellular energy metabolism broadly confirm previously described effects of CMH, but CMH supplementation has also been associated with cytoskeleton remodelling [8]. In the present study, structural perturbations were only indicated by an up regulation of the intermediate filament protein vimentin, which may just reflect maintenance of cellular integrity. Other studies have shown that neither muscle hypertrophy nor performance of rat skeletal muscle was augmented by creatine, and the authors argued that positive findings in relation to performance may rather be due to an enhanced ability to train [34].

Other effects of creatine support the hypothesis of creatine-induced improved ability to train through a direct antioxidant effect of creatine [35] on DNA molecules [36] or through activation of some of the cellular antioxidative systems.

The intracellular protection mechanisms against reactive oxygen species are very delicately balanced and, when exposed to stressors, adjustments in the defense mechanisms may be induced [37]. In various cell cultures including murine myoblasts an increased creatine level was associated with general cytoprotective effects towards oxidative agents [38,39]. However, the activities of the antioxidative enzymes catalase and glutathionperoxidase were not affected by creatine treatment [38,39], and the authors ascribed the cytoprotective effect to scavenging dependent antioxidative mechanisms [38]. In the present study on murine myotubes, we revealed an additional antioxidant effect of creatine, i.e. its capacity to induce up-regulation of one of the cellular antioxidative systems the thiol redox system, which consists of the glutathione and thioredoxin pathways [40]. Two thioredoxin reductases situated in the mitochondria and cytoplasm, respectively, were increased in creatine treated cells (Table 1); peroxiredoxin-4, a type 2 peroxiredoxin, and thioredoxin dependent peroxide reductase. These systems catalyse thiol-disulfide exchange reactions and thereby control the redox state of cytoplasmic cysteine residues, thus protecting e.g. radical sensitive enzymes from oxidative damage. An up-regulation of these very universally important redox systems as well as reduced intracellular DCFH_2 _oxidation (Figure 4) is an indication of an improved resistance towards oxidative challenges in cells exposed to CMH. Improvement of the intracellular antioxidative mechanisms will enhance the ability to cope with the increased levels of reactive oxygen species inevitably following increased exercise.

## Conclusions

CMH supplementation increases the muscle cell energy metabolism, but the direct effect on performance is still debated and investigated. The explorative approach of this study, combined with the finding of a decreased intracellular DCFH_2 _oxidation, revealed an additional stimulation of cellular antioxidative mechanisms when exposed to CMH. This may contribute to an improved performance through increased ability to cope with training-induced increases in oxidative stress. Combined effects of increased energy load and improved antioxidative defences may thus be the key to the performance improvement experienced by some athletes following creatine supplement, but this approach needs further investigation [41].

## List of abbreviations used

CHAPS: 3-[(3-cholamidopropyl) dimethylammonio]-1-propansulfonate; CMH: creatine monohydrate; DCFH_2_: 2',7'dichlorodihydroflourescein; 2-DGE: 2 dimentional gel electrophoresis; DTE: dithioerythritol; GRP75 and GRP78: glucose regulated protein precursors of 75 kDa and 78 kDa, respectively; MALDI-TOF: Matrix Assisted Laser Desorption Ionization - Time of Flight; MS: mass spectrometry; NMR: nuclear magnetic resonance; PLS: partial least squares; PLS-DA: PLS-discriminant analysis; TMSP: sodium trimethylsilyl-[2,2,3,3-^2^H_4_]-1-propionate.

## Declaration of competing interests

The authors declare that they have no competing interests.

## Authors' contributions

JFY and NO participated in the design of the cellular studies and JFY drafted the manuscript. IKS carried out the cellular experiments for proteomics and metabonomics studies. LBL designed the proteomics analysis and HCB, AM and NCN designed and carried out the metabonomics experiments. LBL, HCB and JFY carried out data and statistical analysis on proteomics, metabonomics and DCFH_2 _oxidation analysis, respectively. All authors contributed in the drafting of the manuscript and approved the final manuscript.
